# Effects of the Nanofillers on Physical Properties of Acrylonitrile-Butadiene-Styrene Nanocomposites: Comparison of Graphene Nanoplatelets and Multiwall Carbon Nanotubes

**DOI:** 10.3390/nano8090674

**Published:** 2018-08-29

**Authors:** Sithiprumnea Dul, Alessandro Pegoretti, Luca Fambri

**Affiliations:** Department of Industrial Engineering and INSTM Research Unit, University of Trento, Via Sommarive 9, 38123 Trento, Italy; sithiprumnea.dul@unitn.it (S.D.); alessandro.pegoretti@unitn.it (A.P.)

**Keywords:** mechanical properties, conductive composites, graphene, carbon nanotubes, solvent free compounding

## Abstract

The effects of carbonaceous nanoparticles, such as graphene (GNP) and multiwall carbon nanotube (CNT) on the mechanical and electrical properties of acrylonitrile–butadiene–styrene (ABS) nanocomposites have been investigated. Samples with various filler loadings were produced by solvent free process. Composites ABS/GNP showed higher stiffness, better creep stability and processability, but slightly lower tensile strength and electrical properties (low conductivity) when compared with ABS/CNT nanocomposites. Tensile modulus, tensile strength and creep stability of the nanocomposite, with 6 wt % of GNP, were increased by 47%, 1% and 42%, respectively, while analogous ABS/CNT nanocomposite showed respective values of 23%, 12% and 20%. The electrical percolation threshold was achieved at 7.3 wt % for GNP and 0.9 wt % for CNT. The peculiar behaviour of conductive CNT nanocomposites was also evidenced by the observation of the Joule’s effect after application of voltages of 12 and 24 V. Moreover, comparative parameters encompassing stiffness, melt flow and resistivity were proposed for a comprehensive evaluation of the effects of the fillers.

## 1. Introduction

During recent decades, polymer nanocomposites with carbon-based nanofillers have been extensively investigated for fabrication of multifunctional materials with tailored properties, including high mechanical, thermal and electrical performance. Among these nanofillers, different form of graphene and carbon nanotubes have been commonly utilized due to their extraordinary intrinsic properties [1,2,3,4,5,6,7,8,9,10,11]. The nomenclature of two-dimensional carbon materials is still an object of discussion and confusion, for instance between graphene nanoplatelets or graphite nanoplates. At this purpose, some interesting recommendations are reported by Bianco et al. [12]. In the present study, the authors decided to use the term graphene or graphene nanoplatelets (GNP), in conformity with the commercial name of the product. Various scientific papers on graphene (GNP) or carbon nanotubes (CNT) nanocomposites reported the comparative study of these two nanofillers, detailing their effect in various matrices: epoxy [13,14,15,16,17], polyamide [18] and poly (styrene-b-ethylene-ran-butylene-b-styrene) (SEBS) [19]. GNP and CNT incorporated in epoxy matrix showed marked improvement of mechanical properties, thermal conductivities and dielectric constant of nanocomposites, for example, at the highest concentration of 1 wt % [14,15], 3 wt % [13] and 4 wt % [17] of both fillers. The mechanical and electrical properties of PA/GNP and PA/CNT nanocomposites with 1 wt % nanofiller were compared [18]. Nanocomposites of SEBS matrix filled with single GNP, or CNT, or GNP/CNT mixture at the nanofiller content of 3 wt % up to 15 wt % were produced for electromagnetic shielding applications [19]. Another study was focused on the comparison between graphene and carbon nanotubes in ABS at concentrations up to 1.2 vol % (~2.5 wt %) reached by solution mixing [20]. In this paper, only tensile properties and theoretical model of the modulus of ABS nanocomposites were reported.

One of most frequently used thermoplastics is acrylonitrile-butadiene-styrene (ABS) due to its desirable properties such as easy processing, chemical resistance, toughness, dimensional stability and good surface appearance [21,22]. The processability of ABS can be affected by the presence of the additives such as lubricant, impact modifier, stabilizers and fillers. In particular, moulding lubricant additive significantly decreases the viscosity of ABS, which promotes processability and prevents sticking to processing equipment [23].

Our previous research has been focused on the structure and physical properties of different types of ABS-carbon based nanocomposites. Large lateral size of graphene nanoplatelets in the ABS containing a lubricant provided better mechanical and electromagnetic shielding properties [6]. Two different types of ABS (with and without lubricant additive) GNP filled nanocomposites were compared. The lubricant was found to promote the processability of nanocomposites but slightly reduced the strength of nanocomposites [24]. Subsequently, compositions of ABS/graphene composites were properly selected for fused deposition modelling (FDM). As a result, the improvement of thermo-mechanical properties of ABS/GNP compared to neat ABS matrix was reported [25]. Another our study was focused on conductive ABS nanocomposites (without lubricant) filled CNT for FDM in order to increase the processing shear stresses during CNT dispersion. Highly conductive ABS/CNT nanocomposites were achieved and were successfully 3D-printed at 6 wt % of CNT [7].

The aim of this work is to investigate the effects of GNP and CNT nanofillers in ABS nanocomposites on the physical, mechanical and electrical properties of ABS nanocomposites with the ambition to highlight the potentialities of each nanofiller. Although some prior papers [13,14,15,16,17,18,20] have been dedicated to the comparisons between graphene and CNT nanocomposites with the same ABS matrix, the effect of these two nanofillers on processability, mechanical and electrical properties of nanocomposites filled in the range of 2–8 wt % have not yet been reported. Therefore, the characterization could provide useful information about both CNT and GNP composites, which consequently could be properly selected for specific applications.

In this study, the morphology of the GNP and CNT were observed by using both transmission electron microscope (TEM) and scanning electron microscope (SEM). Stress-strain tests and tensile creep measurements were done on the GNP and CNT filled ABS nanocomposites. The SEM observations were performed on the fracture surface of the both nanocomposites to evaluate the dispersion of fillers. Electrical resistivity and Joule’s effect were also measured. The comparison of between GNP filled ABS nanocomposites and CNT filled ABS nanocomposites is presented and discussed.

## 2. Experimental

### 2.1. Materials

Acrylonitrile-butadiene-styrene (ABS) polymer (tradename Sinkral^®^F322) used in this study was kindly provided by Versalis S.p.A. (Mantova, Italy). According to the producer’s technical data sheet, the polymer is characterized by a density of 1.04 g/cm^3^ and a melt volume rate of 14 cm^3^/10 min (220 °C/10 Kg) [26]. Before processing, ABS chips were dried under vacuum at 80 °C for at least 2 h.

Graphene nanoplatelets (GNP) were purchased from XG Sciences (East Lansing, MI, USA). For the selected type of nanoplatelets (type M5), the manufacturer reports average lateral dimension of 5 μm, a thickness in the range 6–8 nm, a surface area of 120–150 m^2^/g [27].

Carbon nanotubes (CNTs) (tradename NC7000™ multi-walled carbon nanotubes) were provided by Nanocyl S.A. (Sambreville, Belgium). The technical data sheet reports an average length of 1.5 μm, a diameter of 9.5 nm and a surface area of 250–300 m^2^/g [28].

### 2.2. Materials Processing and Sample Preparations

Composites were prepared by melt compounding of ABS with 2–8 wt % GNP or CNT in a co-rotating Thermo-Haake Polylab Rheomix internal mixer (Thermo Haake, Karlsruhe, Germany) at a temperature of 190 °C and a rotor speed of 90 rpm for 15 min, producing about 50 g for each composition. Square plates (160 × 160 × 1.2 mm) of compounded materials and neat ABS matrix were obtained after compression moulding at 190 °C by using a Carver Laboratory press (Carver, Inc., Wabash, IN, USA) for 10 min under a pressure of 3.9 MPa.

### 2.3. Testing Techniques

#### 2.3.1. Transmission Electron Microscopy (TEM)

The morphology of graphene nanoplatelets and carbon nanotubes was observed by transmission electron microscopy (TEM), using a Philips^®^ EM 400 T (Philips, Amsterdam, The Netherlands) transmission electron microscope at an acceleration voltage of 120 kV. Nanoparticles were dispersed in acetone and the suspension (concentration = 0.5 mg/mL) was sonicated for 5 min. Afterwards, the nanoparticle suspensions were dropped on a 600-mesh copper grid for TEM observation.

#### 2.3.2. Scanning Electron Microscopy (SEM)

Nanocomposites were fractured in liquid nitrogen and fracture surfaces were observed by using a Carl Zeiss AG Supra 40 field emission scanning electron microscope (FESEM) (Carl Zeiss AG, Oberkochen, Germany) at an acceleration voltage of 3 kV.

#### 2.3.3. Melt Flow Index

Melt flow index (MFI) measurements were carried out in a temperature range of 220–280 °C with an applied load of 10 kg by using a capillary rheometer Kayeness Co. model 4003DE (Morgantown, PA, USA) following ASTM D 1238 standard (procedure A). Samples of about 5 g were loaded in the cylinder and pre-heated for about 5 min before testing. The results are reported in Table 1 as average values of at least five measurements (standard deviation is reported).

#### 2.3.4. Quasi-Static Tensile Test

Uniaxial tensile tests were performed at room temperature on ISO 527 type 1BA specimens by using an Instron^®^ 5969 universal testing machine (Norwood, MA, USA), equipped with a 50 kN load cell. Test specimens were die-cut from compression moulded plates (gauge length of 30 mm; width of 5 mm; thickness of 1.2 mm). Elastic modulus was determined at a crosshead speed of 1 mm/min with the secant method between strain levels of 0.05% and 0.25% according to ISO 527 standard and by using an electrical extensometer Instron^®^ Model 2620-601 (Norwood, MA, USA) with gauge length of 12.5 mm for strain monitoring. Yield stress (*σ_y_*), stress at break (*σ_b_*) and strain at break (*ε_b_*), were evaluated at a crosshead speed of 10 mm/min without extensometer. Specific tensile energy to break (TEB) values under quasi-static conditions were computed integrating stress-strain curves. The results are reported in Table 2 as average values of at least five specimens (standard deviation is reported).

#### 2.3.5. Creep Test

A creep test was performed with the aid of TA Instruments DMA Q800 (TA Instruments-Waters LLC, New Castle, DE, USA) under a constant stress of 3.9 MPa (i.e., about 10% of the yield stress of neat ABS) at 30 °C up to 3600 s. Rectangular samples with length of 25 mm, width of 5 mm and thickness of 0.9 mm were machined from compression moulded plaques. The adopted gauge length of all samples was 11.8 mm.

#### 2.3.6. Electrical Resistivity and Resistive Heating

For samples with electrical resistivity higher than 10^7^ Ω·cm, the volume resistivity was measured according to the ASTM D257 by using a Keithley 6517A electrometer/High Resistance Meter (Beaverton, OR, USA) and an 8009 Resistivity Test Fixture at the room temperature. In this test, a constant applied voltage of 100 V was applied to a square specimen of 64 × 64 mm.

For moderately conductive materials (<10^7^ Ω·cm), volume electrical resistivity was determined following ASTM D4496-04 standard for moderately conductive materials with four-point contact configuration. Each specimen was tested at a voltage of 5 V by using a direct current (DC) power supply IPS303DD produced by ISO-TECH (Milan, Italy) and the current flow on the samples was measured between external electrodes using an ISO-TECH IDM 67 Pocket Multimeter electrometer (ISO-TECH, Milan, Italy). Compression moulded samples were tested with a length of 25 mm and different cross-section (rectangular specimens 6 × 1.2 mm). At least three specimens were replicated for each sample. The electrical volume resistivity of the samples was evaluated by Equation (1):

(1) $$\rho =R\times \frac{A}{L}\;$$

where *R* is the electrical resistance, *A* is the is the cross-section of the specimen and *L* is the distance between the internal electrodes (i.e., 3.69 mm).

The heating of a sample generated by current flow is known as resistive heating and it is described by the Joule’s law. Surface temperature evolution induced by Joule’s effect upon different applied voltages was measured by a Flir E6 thermographic camera (FLIR System, Wilsonville, OR, USA). The voltages were applied by a DC power supply (IPS 303DD produced by ISO-TECH), while the samples were fixed with two metal clips with an external distance of 30 mm. In these tests, specimen length was 50 mm with different cross-sections of rectangular 6 × 1.2 mm. The surface temperature values have been recorded for 120 s of application of the voltage levels of 12 V and 24 V. These voltages have been selected because they are common voltages for batteries used in automotive, solar storage equipment, electrical bikes and other domestic applications. Due to high conductivity, only the Joule’s effect of the ABS/CNT nanocomposites was reported.

## 3. Results and Discussions

### 3.1. Morphology

**Figure 1:**
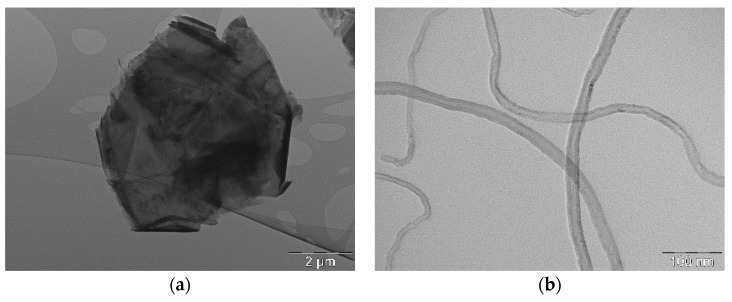
TEM micrographs of the selected carbonaceous nanoparticles: (**a**) GNP and (**b**) CNT.

The morphologies of graphene (GNP) and carbon nanotube (CNT) have been characterized by TEM microscopy (Figure 1). Figure 1a shows the typical thin sheet structure of GNP. From TEM micrographs, the average diameter of platelets of GNP has been found to be about 5.5 to 6.8 μm. In addition, it was also observed that some GNP nanoplatelets superimposed on top of each other and wrinkled into an irregular shape. Figure 1b displays the morphological structure of CNT and clearly documents that the investigated CNT have the outer diameter of tubes about 15–20 nm with wall thickness of about 4–6 nm.

**Figure 2:**
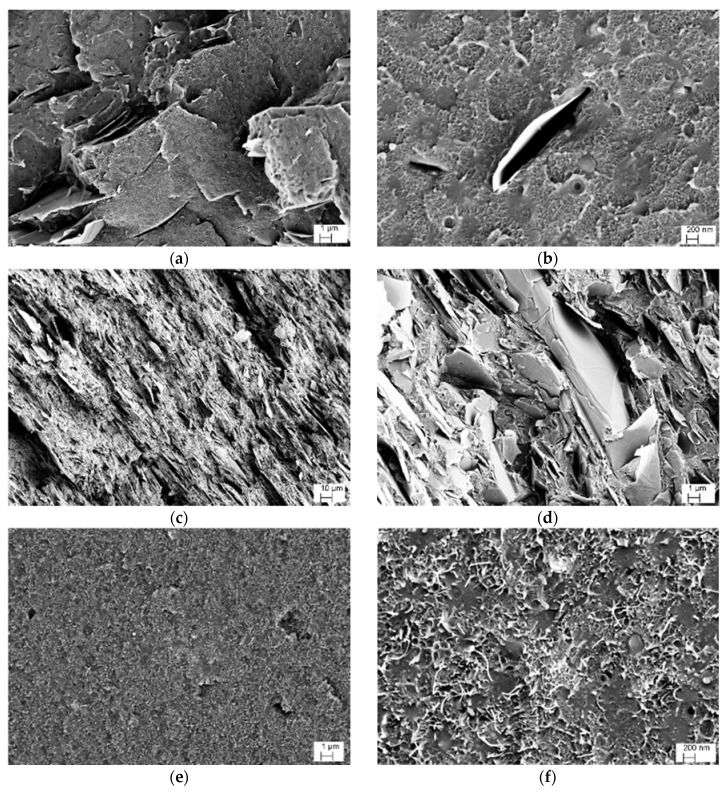
SEM micrographs of the samples of GNP-6 (**a**,**b**), GNP-30 (**c**,**d**) and CNT-6 (**e**,**f**).

The SEM images of the fracture surfaces of ABS/graphene and ABS/CNT samples are represented in Figure 2a–d and Figure 2e–f, respectively. A relatively poor adhesion level between graphene and ABS is documented in Figure 2b. Figure 2c shows GNP-30 sample with the highest GNP concentration where graphene flakes appear distributed quite evenly within the matrix up to the highest concentration of 30 wt %. A relatively good dispersion was also observed for graphene nanoplatelets. As it can be seen in Figure 2e–f, carbon nanotubes were clearly observed in the SEM micrographs with uniform distribution and excellent dispersion.

### 3.2. Melt Flow Index

**Figure 3:**
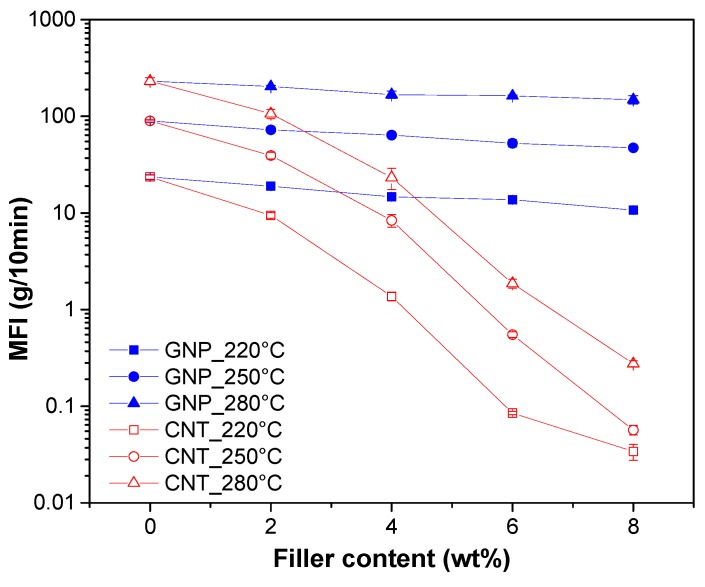
Melt flow index of ABS/graphene (full symbols) and ABS/CNT (open symbols) nanocomposites at selected temperatures and nanofiller fractions.

The processability of the nanocomposite materials was investigated by comparing their melt flow index values. Figure 3 summarizes that the effect of the nanofiller amounts, the types of nanofiller and the temperature on MFI value of nanocomposites. As expected, the MFI values decreased with the nanofiller fraction. The MFI values of various compositions at 220, 250 and 280 °C are reported in Table 1). It is worthwhile to note that CNT accounts for a much higher reduction in MFI than GNP, thus temperature of 220–250 °C does not allow satisfactory conditions for materials processing. Therefore, 280 °C has been found as an adequate temperature for processing ABS/CNT composites with 6–8% of CNT.

**Figure 4:**
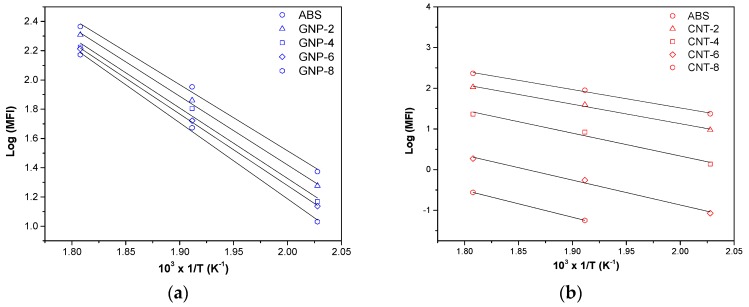
Melt flow index as a function of temperature of the composites with (**a**) graphene and (**b**) carbon nanotubes.

Moreover, following the results of MFI at different temperature, the activation energy (*E_act_*) for polymer chain mobility in both ABS and nanocomposites could be evaluated from the slope of the best fitting straight lines (Figure 4) by using an Arrhenius type equation [29,30]:

(2) $$Log(\mathrm{MFI})=Log(C_{0})-\left(\frac{E_{act\;}}{2.303R}\right)1/T$$

where *C*_0_ is a pre-exponential factor, *T* is the selected temperature for MFI test and *R*, the universal gas constant, is 8.314 J/mol·K. The value of intercept *C*_0_ formally represents MFI at infinite temperature.

As reported in Table 1, the activation energy of neat ABS is about 87 kJ/mol. As expected, the higher the filler content, the lower the polymer chain mobility and, consequently, the higher the activation energy of the process flow. In particular, it should be underlined that the activation energy of ABS/CTN nanocomposites is higher than that of corresponding graphene nanocomposites. Thus, the higher activation energy could indicate a stronger of interaction between CNT and ABS in the molten state. Consequently, CNT nanocomposites require more energy for processing which is documented by the fact that the flow activation energy of nanocomposite. For instance, *E_act_* of 6 wt % of nanofiller is about 94 kJ/mol and 117 kJ/mol for GNP and CNT, respectively. The difference is even higher in composites at 8 wt % (see Table 1).

### 3.3. Quasi-Static Tensile Test

Tensile testing was carried out to investigate the reinforcing effect of graphene and CNT in ABS nanocomposites. Stress-strain curves are reported in Supplementary Materials (Figure S1), whereas the tensile properties of ABS/GNP and ABS/CNT nanocomposites are summarised in Table 2. As expected, both ABS/GNP and ABS/CNT show an enhancement of tensile properties compared to neat ABS. In Table 2, the elastic modulus of GNP-based nanocomposites is higher than that of CNT-based composites. For instance, the elastic modulus of composites containing 8 wt % of GNP was increased from 2315 MPa to 3523 MPa (i.e., 52%) whereas in the case of 8 wt % of CNT a corresponding value is only 3068 MPa (i.e., 32%). The elastic modulus of the composites is affected by nanofiller stiffness but also by the shape and orientation of particles and their dispersion level. Yielding phenomenon was observed only for nanocomposites containing less than 4%.

On the other hand, various factors affecting the tensile strength of ABS nanocomposites include the filler/matrix interfacial adhesion, the amount of filler, its properties and geometry and dispersion level in the matrix. Table 2, shows that strength of ABS/CNT composites is higher than that of ABS/GNP nanocomposites which can be attributed to the better dispersion and interaction of CNT with ABS matrix with respect to GNP, as shown in Figure 2c–f. Moreover, this result could be associated with the two-dimensional (2D) shape of GNP which makes plane-to-plane contact area and could to be wrinkled and be detached from ABS (see Table 2). On the other hand, some bending and twisting in the structure of the CNT could prevent the detachment of CNT from ABS matrix. Thus, these factors may induce a better interfacial interaction between the CNT and ABS matrix. As a result, the load can efficiently be transferred from the ABS matrix to CNT nanofillers and therefore the tensile strength is higher for CNT nanocomposites. Analogously, the strain at break was observed to be more severely reduced in the case of GNP nanocomposites.

Another interesting result is the possibility to achieve very high concentrations of GNP by proper processing conditions. In particular, the 30 wt % reported in this present paper represent the highest fraction reported in the literature for ABS/graphene composites. Interestingly enough, these composites show elastic modulus of about 7362 MPa and tensile strength of about 44 MPa.

To compare the mechanical properties of ABS composites reported in literature, a normalized modulus was evaluated as follows:

(3) $$\mathrm{Normalized}\;\mathrm{modulus}=E_{norm\;}=\frac{E_{c}-E_{m}}{E_{m}w_{f}}$$

where *E_c_* is the modulus of ABS composite; *E_m_* is the matrix modulus of neat ABS and *w_f_* is the weight fraction of incorporated filler [6,31].

According to the experimental data, a maximum strength value was obtained for 6 wt % of CNT, whereas a maximum stiffening effect (*E_norm_*) was observed for 6 wt % of GNP, maintaining an acceptable deformation at break (3–4%) for both the compositions, even in absence of yielding.

The empirical Halpin-Tsai model is a simple approach to predicting the modulus of composite materials which takes into account the modulus of matrix *E_M_* and filler *E_F_*, filler aspect ratio *ξ*, the volume fraction of filler *V_f_*, assuming a homogeneous dispersion and perfect interfacial adhesion between polymer/filler [32,33,34,35,36]. The tensile modulus in both longitudinal *E_L_* and transverse *E_T_* directions can be predicted according to Halpin-Tsai model [37,38] by the following equations:

(4) $$E_{L}=\frac{1+\xi \eta_{L}V_{f}\;}{1-\eta_{L}V_{f}}E_{M}$$

(5) $$E_{T}=\frac{1+2\eta_{T}V_{f}\;}{1-n_{T}V_{f}}E_{M}$$

where the parameters *η_L_*, *η_T_* and *ξ* are defined as [34,39,40]:

(6) $$\eta_{L}=\frac{(E_{f}/E_{M})-1\;}{(E_{f}/E_{M})+\xi }$$

(7) $$\eta_{T}=\frac{(E_{f}/E_{M})-1\;}{(E_{f}/E_{M})+2}$$

(8) $$\xi =\frac{2}{3}\frac{D_{f}}{t_{f}}\;\mathrm{for}\;\mathrm{plates}$$

(9) $$\xi =2\frac{L_{f}}{D_{f}}\;\mathrm{for}\;\mathrm{fibres}$$

*D_f_* and *t_f_* are lateral diameter and thickness of platelets and *L_f_* and *D_f_* are length and diameter of fibres, respectively.

The volume fraction *V_f_* is linked to the weight fraction *w_f_* through the following equation:

(10) $$V_{f}=\frac{w_{f}\rho_{M}\;}{w_{f}\rho_{M}+(1-w_{f})\rho_{f}}$$

where, *ρ*_M_ and *ρ*_f_ are the densities of the ABS matrix and graphene nanoplatelets, respectively.

Subsequently, the modulus of a composite with platelet filler along the axis parallel to the loading direction (*E^Parallel^*) and randomly oriented platelets/fibres fillers in all two dimensional 2D-direction (*E*^2*D*,*Random*^) and three-dimensional 3D-directions (*E*^3*D*,*Random*^) can be predicted according to literature [39,40,41] as follows.

For plates:

(11) $$E_{c}^{Parallel\;}=E_{L}$$

(12) $$E_{c}^{3D,Random\;}=0.49E_{L}+0.51E_{T}$$

For fibres:

(13) $$E_{c}^{Parallel\;}=E_{L}$$

(14) $$E_{c}^{2D,Random\;}=0.375E_{L}+0.625E_{T}$$

(15) $$E_{c}^{3D,Random\;}=0.184E_{L}+0.816E_{T}$$

In the Halpin-Tsai model an experimental modulus for neat ABS of 2315 MPa was considered (Table 2). The aspect ratios are considered to be equal to 833 for GNP (*D_f_* = 5000 nm and *t_f_* = 6 nm) and 158 for CNT (*L_f_* = 1500 nm and *D_f_* = 9.5 nm). An elastic modulus of 70 GPa has been assumed for both graphene and carbon nanotubes [42,43].

**Figure 5:**
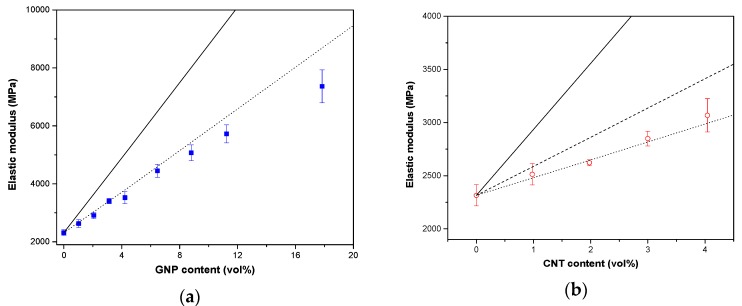
Elastic modulus of nanocomposites ABS/GNP (**a**) and ABS/CNT (**b**), evidenced in blue and red symbols, respectively. Continuous (^___^) and dash lines (^_ _ _^) and dot lines (···) represent prediction according to the Halpin-Tsai models with parallel, 2D random and 3D random orientation, respectively.

The experimental data show good agreement with Halpin-Tsai model assuming a 3D randomly oriented nanofiller due to the melt compounding process. However, the elastic moduli of ABS/GNP nanocomposites were lower than the predicted elastic modulus when the content of GNP is higher than 6.5 vol % (12 wt %), which indicates a decrease in the reinforcing efficiency (see Figure 5b).

### 3.4. Creep Stability

**Figure 6:**
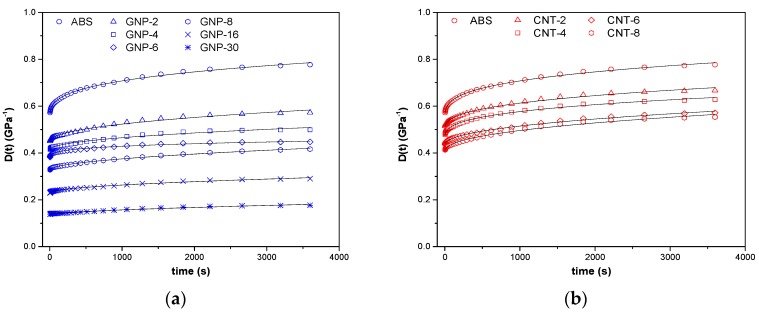
Creep compliance of nanocomposites with (**a**) graphene and (**b**) carbon nanotubes at 30 °C at 3.9 MPa.

The isothermal creep compliance of ABS/GNP and ABS/CNT nanocomposites under a constant load of 3.9 MPa and at 30 °C is reported in Figure 6a,b. If no plastic deformation occurs, compliance of isothermal tensile creep, *D_tot_*(*t*), consists of two components: elastic (instantaneous) *D_el_* and viscoelastic (time-dependent) *D_ve_*, as defined in Equation (16).

*D_tot_(t) = D_el_ + D_ve_(t)* (16)

Following the described models of creep evaluation, the elastic (*D_e_*), viscoelastic *D_ve_*_,3600s_ and total (*D_t_*_,3600s_) components of the creep compliance after 3600 s are have been calculated; the results are summarized in Table 3. As expected, the introduction of graphene or carbon nanotubes results in a significant improvement of the creep stability of the materials. As expected, the higher the filler content, the lower the creep compliance (see Table 3). The role of nanofillers is to restrict the polymeric chain mobility, thus increasing creep stability. The empirical Findley’s model (power law), summarized in Equation (17), was used to describe the viscoelastic creep response [44,45,46]:

*D(t) = D_e_ + kt^n^* (17)

where *D_e_* is the elastic (instantaneous) creep compliance, *k* is a coefficient related to the magnitude of the underlying retardation process and n is an exponent related to the time dependence of the creep process. The fitting parameters for experimental creep data are summarized in Table 3. The fitting model was satisfactory, as R^2^ around 0.99 was found for all samples. The coefficient n reflects, which the kinetics of displacements of the segments of macromolecules in the viscous medium in the course of the creep. ABS/GNP exhibit n value higher than correspondent ABS/CNT nanocomposites.

In addition, the creep compliance of GNP nanocomposite appeared to be significantly lower than that of reduced with respect to CNT nanocomposite at the same nanofiller fraction. This reduction is largely associated with the reduction of the values of elastic component (either *D_el_* or *D_e_*, for both the models). In summary, ABS/graphene nanocomposites exhibited a higher creep stability than CNT nanocomposites, which in agreement with observed difference of moduli (as shown in Table 2).

**Figure 7:**
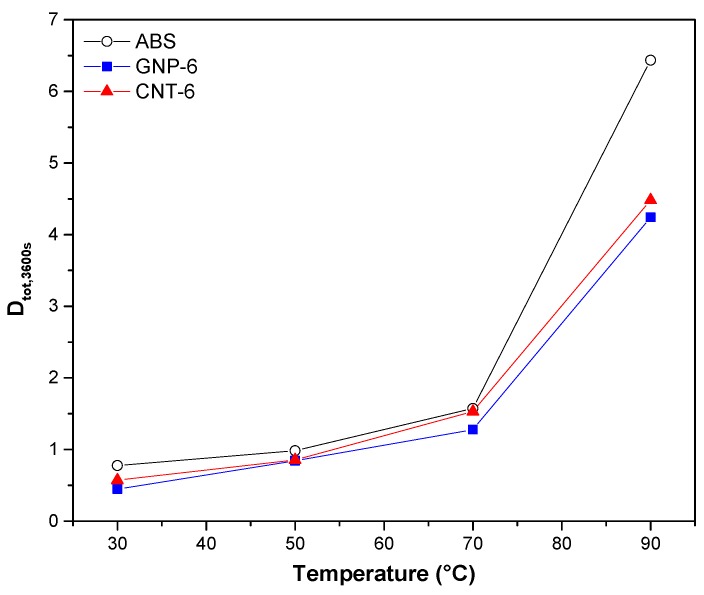
Creep compliance (*D_tot_*_,3600s_) of neat ABS, GNP-6 and CNT-6 nanocomposites at 3.9 MPa at various temperatures in the range of measurements.

Further information can be obtained by considering the creep compliance curves at various temperatures from 30 °C to 90 °C (see Figure S2 and Table 4). It can be noted that the higher temperature the high creep compliance especially for neat ABS in proximity of *T_g_*. Selected data are shown in Figure 7; a relevant reduction of the creep compliance of GNP-6 and CNT-6 was observed at the highest investigated temperature (90 °C).

From the results of total creep compliance at different temperatures, activation energy (*E_act_*) of the creep process for the investigated nanocomposites can be evaluated from the slope of the best fitting straight lines by using an Arrhenius type equation, as previously [29,30]:

(18) $$Log(D_{t,3600s\;})=Log(D_{0})-\left(\frac{E_{act}}{2.303R}\right)1/T$$

where *D*_0_ is a pre-exponential factor, *T* is the selected temperature for creep experiment and *R*, the universal gas constant, is 8.314 J/mol·K. The value of intercept *D*_0_ formally represents creep compliance at infinite temperature.

In Table 5, the activation energy of neat ABS is 15 kJ/mol and it increases for nanocomposite with 6 wt % of graphene or CNT. It should be noted that the activation energy of ABS/GNP nanocomposites appears to be a little bit higher than that of corresponding ABS/CNT nanocomposites which may explain higher creep stability.

### 3.5. Electrical Behaviour

#### 3.5.1. Electrical Resistivity

**Figure 8:**
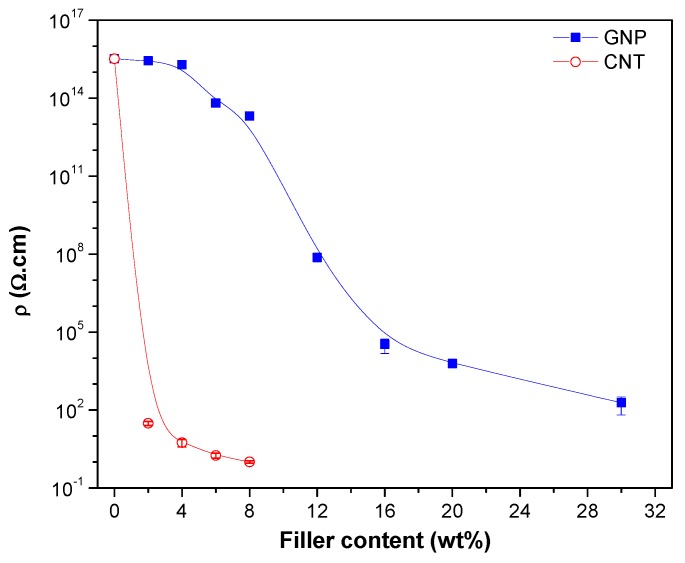
Electrical volume resistivity of ABS/GNP and ABS/CNT nanocomposites. The applied voltage was 5 V or 100 V for samples having resistivity lower or higher than 10^7^ Ω·cm, respectively.

The electrical volume resistivity values of CTN or graphene filled ABS (compression moulded) plates are reported in Figure 8 as a function of the nanofiller fraction. The introduction of the carbon-based nanofiller in the insulating polymeric matrix increases the conductivity of the nanocomposites with direct dependence on the type and the content of nanofiller. For example, a resistivity value lower than 10^2^ Ω·cm can be achieved with CNT content of 2 wt %. The introduction of CNTs confers a good conductivity to the nanocomposites samples, which makes possible to evaluate the lower electrical percolation threshold in CNT nanocomposites with respect to GNP-filled nanocomposites. This threshold value is below 2 wt % for CNT while values between 8 and 12 wt % were found for GNP. Higher resistivity reduction reached with the introduction of carbon nanotube could be attributed to the better dispersion level.

According to the statistical percolation theory, the data of electrical resistivity as a function of filler volume fraction can be fitted by a power law equation:

(19) $$\sigma =\sigma_{o}{(\phi -\phi_{c})\;}^{t}$$

The equation can be adapted to electrical resistivity as follows:

(20) $$\log(\frac{1}{\rho })=t\log(\phi -\phi_{c})+\log({\frac{1}{\rho }\;}_{o})$$

where *ρ* = composites resistivity, *ρ*_o_ = scale factor related to the filler intrinsic resistivity, ϕ = filler volume fraction, $\phi_{c}$ = percolation threshold and *t* = critical exponent. Exponent *t* value in the range 1.1–1.3 indicates the conduction through 2D network whereas for 3D network the value lies in the range of 1.6–2.0.

**Figure 9:**
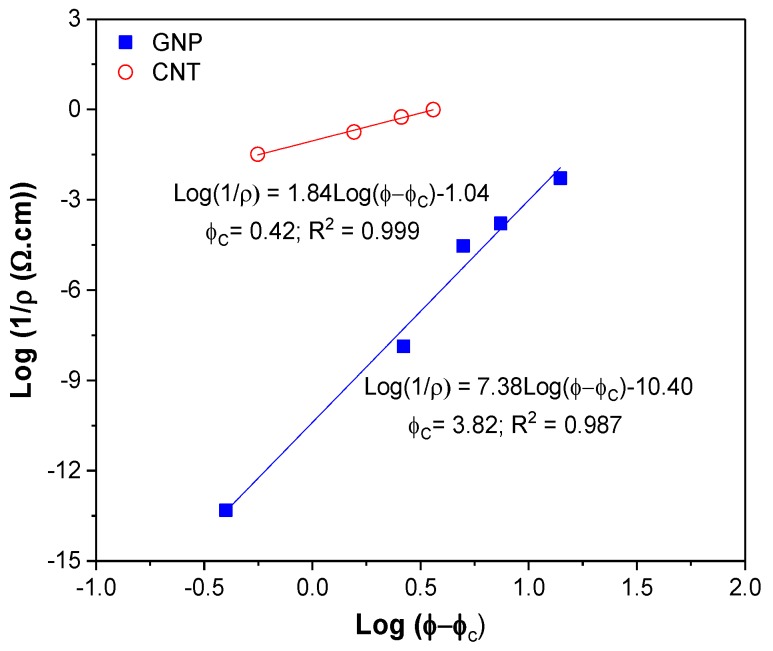
Resistivity of composites as function of filler volume fraction and calculated percolation threshold according to power law fit from Equation (20).

The best-fit line in Figure 9 shows that the percolation threshold is 3.8 vol % (~7.3 wt %) for GNP and 0.4 vol % (~0.9 wt %) for CNT, respectively. In addition, the *t* values were found to be 7.3 and 1.8 for graphene and carbon nanotubes, respectively. The results suggested that a 3D network is formed in both GNP and CNT composites. In literature, Zhao et al. [47] reported *t* values in the range of 2.40–6.92 for graphene-based polymer composites. The *t* values for CNT were found in the range of 1.3–4.0 [48] and around 2.0 [49]. Various models applying different parameters for the evaluation of conductivity and other properties have also been presented in a recent paper by Zare et al. and in other references therein [50].

#### 3.5.2. Surface Temperature under Applied Voltage

In this section, the measurements of Joule’s heating produced by voltage application on the samples with different contents of CNT are presented. These tests were performed by using two different voltages, 12 V and 24 V which are commonly reached by batteries for automotive applications. We monitored the evolution of the surface temperature of studied nanocomposites as a function of applied voltage and exposition time.

**Figure 10:**
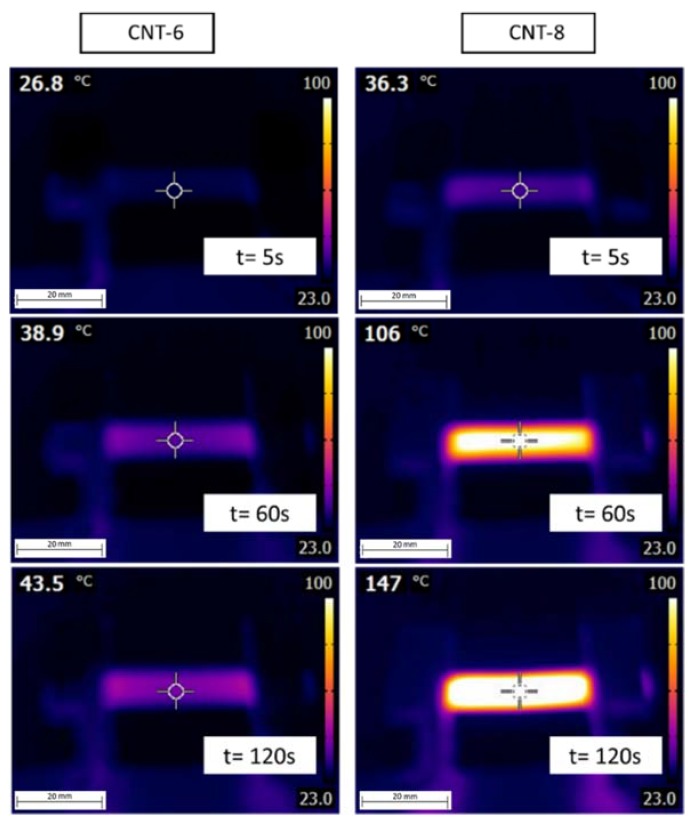
Infrared thermal imaging of CNT-6 (**left**) and CNT-8 (**right**) nanocomposites samples under an applied voltage of 12 V.

**Figure 11:**
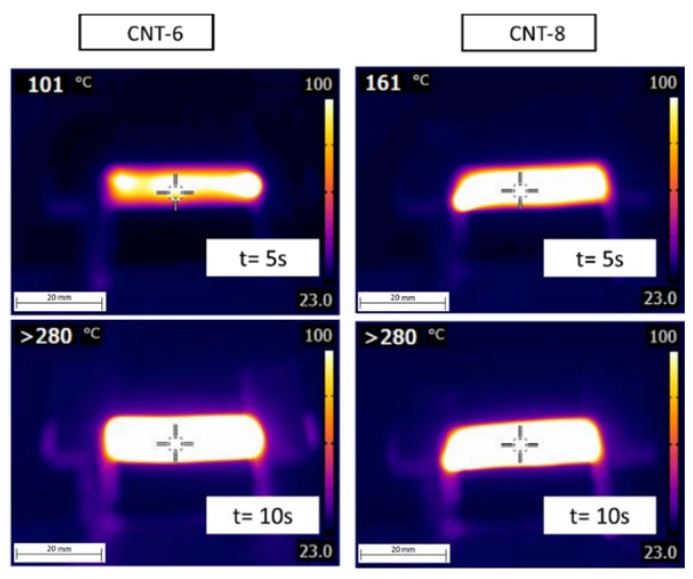
Infrared thermal imaging of CNT-6 (**left**) and CNT-8 (**right**) nanocomposites samples under an applied voltage of 24 V.

Representative images of the surface temperature evolution taken by an IR thermocamera under an applied voltage of 12 V for the CNT-6 and CNT-8 nanocomposites are reported in Figure 10. It is evident that the samples start heating as soon as a voltage is applied and a homogeneous temperature profile can be detected even after prolonged time (i.e., 120 s). As it could be expected, the temperature in the central section of the sample is higher than that detectable on the borders, because the heat exchange is favoured in the external zones of the samples. Under an applied voltage of 24 V (see Figure 11), both CNT-6 and CNT-8 samples rapidly reached a temperature higher than 280 °C after 10 s. On the other hand, graphene composites of 2–8 wt % nanofiller fraction are below the percolation threshold (*ρ* > 10^13^ Ω·cm) and consequently no heating is generated on samples.

**Figure 12:**
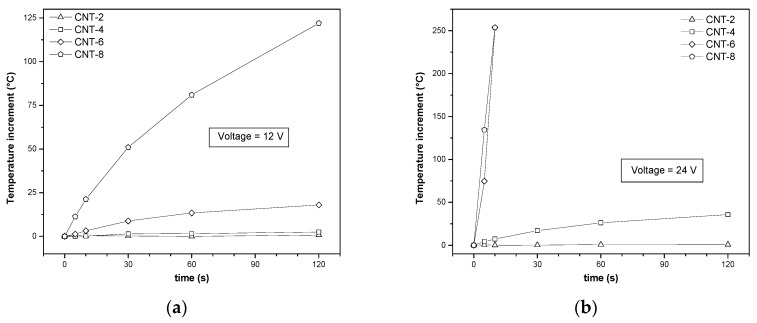
Increment of surface temperature as a function of time for voltage of 12 V (**a**) and 24 V (**b**) applied to ABS/CNT nanocomposites with different fractions of CNT content (starting temperature of 23 °C).

The numerical results of the temperature increment upon an applied voltage of 12 V and 24 V on samples are shown in Figure 12a,b, respectively. The first aspect to underline is that not all samples can be significantly heated through the voltage application. In fact, only samples with CNT content higher than 4 wt % can increase their surface temperature when a voltage of 12 V is applied. At applied voltage of 24 V, only CNT-2 sample does not significantly increase its surface temperature, while CNT-4 sample shows a moderate heating after 120 s. Very effective results can be obtained for all the other samples. For instance, for CNT-6 and CNT-8 samples, it was not possible to reach the end of the test because they thermally decompose with the emission of dense smoke, characteristic of polymers containing aromatic rings.

### 3.6. Comparative Effects of GNP and CNT

**Figure 13:**
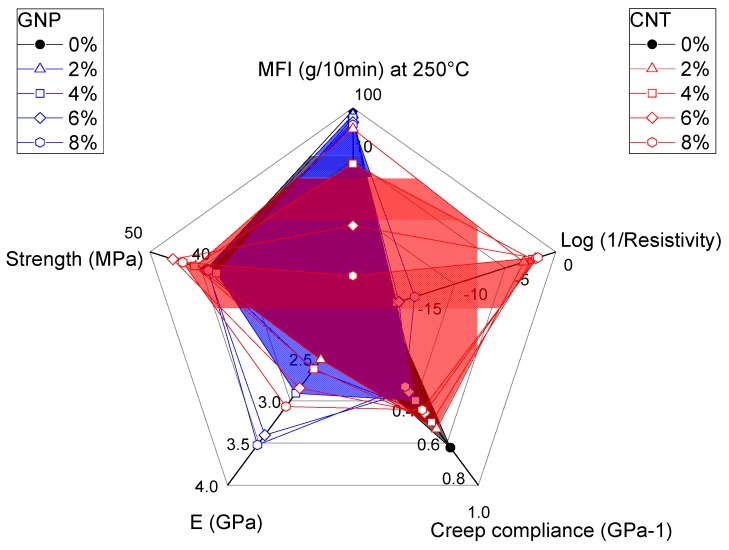
Comparison of selected properties of ABS/GNP and ABS/CNT nanocomposites as function of nanofiller fraction (2–8 wt %).

In order to evaluate beneficial or negative effects of GNP and CNT on the properties of ABS nanocomposites, a graphical representation of selected properties is given in a radar plot. Figure 13 clearly evidences that GNP exhibits positive effect on mechanical properties, that is, increases tensile modulus and reduces creep compliance. In the same time, only small reductions of MFI and of tensile strength were observed. On the other hand, CNTs account for an interesting and valuable improvement of conductivity (i.e., reduction of resistivity) and slight increase in tensile strength but on the other hands deteriorates material processing due to high reduction of melt flow.

Hence it is possible to conclude that both GNP and CNT simultaneously have positive and negative effects on processing as well as properties of the nanocomposites, even if at different levels. 

For the purpose of quantitative evaluation of the nanofiller effects we can use the definition of comparative parameters that take into consideration some specific properties important for of the applications. A first parameter *P_E_*_,_*_ρ_* that maximizes both the stiffness and the conductivity can be calculated from Equation (21)

*P_E_*_,_*_ρ_* = *E*/*ρ* (21)

where *E* and *ρ* represent the modulus and the resistivity of ABS and its nanocomposites, respectively. The comparison of these parameters is reported in Figure S3. CNT nanocomposites evidenced the higher parameters, where the predominant factor is their higher conductivity and *P_E_*_,_*_ρ_* of CNT nanocomposites were found to directly increase with the filler fraction. On the other hand, much lower values were observed for GNP nanocomposites, with only a small variation between 2–4% and 6–8 wt % of filler content.

**Figure 14:**
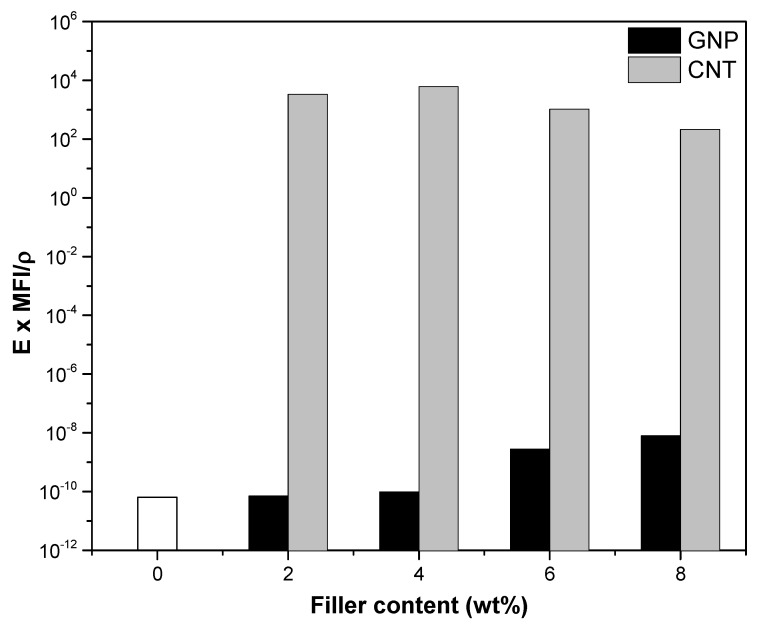
Comparison of parameters P_E,M,_***_ρ_*** (250 °C) combining the effects of elastic modulus, melt flow index at 250 °C and resistivity as a function of nanofiller fraction up to 8 wt %.

However, for a better comparison of nanofillers, the nanocomposites processability should be also taken into account. Hence, an interesting parameter *P_E_*_,*M*,_*_ρ_* defined by Equation (22) can be used for the comparison:

*P_E_*_,*M*,_*_ρ_* = *E* × MFI/*ρ* (22)

where MFI is the melt flow index. Figure 14 shows that only 6 and 8% of GNP lead to a positive variation of the combined factors, that is, stiffness, processability and resistivity of composites with respect to ABS matrix. As can be seen, the parameter *P_E_*_,*M*,***ρ***_ is much higher for CNT nanocomposites than for GNP composites and remains almost constant for all the investigated compositions in the range 2–8% of CNT. In general, the values of *E* × MFI/*ρ* increased with filler fraction for nanocomposites with 6–8% of GNP, while a relative maximum was reached for nanocomposites at 4 wt % of CNT.

Similarly, the same comparative parameter *P_E_*_,*M*,_*_ρ_* was also evaluated as a function of MFI at 220 °C. Figure S4 covers the regions up to the highest experimental fraction of the filler, that is, 8 wt % of CNT and 30 wt % of GNP in ABS nanocomposites. From this point of view, the effect of 20–30% of GNP is approximately equivalent to that of 6–8% of CNT.

Some more detailed comparison of the tested properties is reported in Supplementary Materials Table S1. Obviously, proper combination of these two fillers can be selected in order to tune the properties of the ABS/GNP/CNT nanocomposites for the intended applications.

## 4. Conclusions

Using a solvent free mixing process, GNP and CNT nanofillers were properly dispersed in ABS matrix up to their maximum concentration, that is, 30% and 8% by wt, respectively. Comparative experimental results evidence that the incorporation of both nanofillers produced significant effects on the properties of the ABS nanocomposites.

The addition of nanofillers accounts for an increase in modulus and tensile strength as well as significant reduction of the strain at break. In particular, ABS/GNP composites show a slightly higher stiffness and creep stability than ABS/CNT ones. On the other hand, the tensile strength of ABS/GNP samples up to 20% of the filler was similar to that of neat ABS, whereas the tensile strength of the ABS/CNT composites was slightly enhanced, probably due to better dispersion level and stronger interfacial interactions between CNTs and the ABS matrix. Furthermore, ABS/GNP nanocomposites with 30 wt % filler content showed higher elastic modulus and strength, that is, of 7.4 GPa and 44 MPa, still maintaining moderate processability. On the other hand, significant or almost critical reduction of MFI was observed with rising fraction of CNT. For this reason, CNT nanocomposites need to be processed at higher temperatures than corresponding GNP nanocomposites.

ABS/CNT nanocomposites showed much lower electrical resistivity than corresponding ABS/GNP composition. In fact, a marked resistivity drop in the range 1–10 Ω·cm was observed after dispersion of 4–8 wt % of CNT. Electrical percolation threshold was achieved for 7.3 wt % (3.8 vol %) of GNP and 0.9 wt % (0.4 vol %) of CNT. In the case of CNT-filled samples, the Joule’s effect tests indicated a rapid heating upon voltage application. In contrast, no increase in the temperature during prolonged voltage application was observed for ABS/GNP nanocomposites. It is possible to conclude that the investigated carbonaceous nanofillers account for positive as well as some negative effects on tested physical properties of ABS composites. It is worth noting that GNP brings about notable increases in modulus and only rather moderate reduction of melt flow index. In the case of CNT, the relevant improvement of nanocomposite conductivity is accompanied by a marked increase of melt viscosity, which could be problematic in material processing.

For future work, it can be presumed that proper combinations of CNT and GNP at convenient fractions (e.g., 6 wt % in the hybrid) can offer a possible compromise including relatively easy processability, acceptable mechanical properties and specific electrical properties for applications which require polymeric materials with low electrical resistivity.

## Figures and Tables

**Table 1:** Melt flow index (10 Kg, various temperatures) and activation energy for neat ABS and nanocomposites with graphene or carbon nanotubes.

Samples	MFI (g/10 min)			Log(C_0_)	*E_act_*^1^ (kJ/mol)
	220 °C	250 °C	280 °C		
ABS	23.6 ± 1.3	89.7 ± 2.5	232 ± 19	10.6 ± 0.6	86.6 ± 5.5
GNP-2	18.9 ± 1.0	72.5 ± 1.8	203 ± 6	10.8 ± 0.3	89.9 ± 3.9
GNP-4	14.7 ± 1.0	63.9 ± 0.7	172 ± 5	11.0 ± 0.7	93.1 ± 7.3
GNP-6	13.7 ± 0.7	52.7 ± 4.3	163 ± 4	11.1 ± 0.2	93.7 ± 1.6
GNP-8	10.7 ± 0.5	47.1 ± 1.0	149 ± 14	11.6 ± 0.4	99.6 ± 3.9
GNP-12	8.3 ± 0.2	nt ^2^	nt ^2^	nt ^2^	nt ^2^
GNP-16	6.3 ± 0.2	nt ^2^	nt ^2^	nt ^2^	nt ^3^
GNP-20	5.2 ± 0.4	nt ^2^	nt ^2^	nt ^2^	nt ^3^
GNP-30	1.9 ± 0.1	nt ^2^	nt ^2^	nt ^2^	nt ^2^
CNT-2	9.5 ± 0.6	39.3 ± 3.2	107 ± 12	10.7 ± 0.6	91.7 ± 6.3
CNT-4	1.4 ± 0.1	8.4 ± 1.3	23.3 ± 5.8	11.6 ± 1.4	107.6 ± 13.6
CNT-6	0.08 ± 0.01	0.55 ± 0.04	1.87 ± 0.19	11.4 ± 1.0	117.2 ± 10.3
CNT-8	0.03 ± 0.01	0.06 ± 0.01	0.28 ± 0.02	11.4	126.8 ^3^

^1^*E_act_* activation energy was evaluated according to Equation (2); ^2^ nt = not tested; ^3^ evaluated in the range temperature of 250 °C and 280 °C.

**Table 2:** Comparison of the tensile properties of ABS/CNT and ABS/GNP nanocomposites.

Samples	*E* (MPa)	*σ_y_* (MPa)	*σ_b_* (MPa)	*ε_b_* (%)	TEB(MJ·mm^−3^)	*E_norm_* ^1^
ABS	2315 ± 100	41.7 ± 0.4	33.6 ± 0.4	35.9 ± 6.1	11.785 ± 2.007	nd ^2^
GNP-2	2631 ± 133	41.5 ± 1.2	39.9 ± 2.3	4.1 ± 0.2	1.057 ± 0.078	6.8
GNP-4	2911 ± 109	40.2 ± 1.5	39.3 ± 1.2	3.7 ± 0.2	0.929 ± 0.096	6.4
GNP-6	3406 ± 86	-	41.5 ± 0.8	3.1 ± 0.1	0.788 ± 0.086	7.9
GNP-8	3523 ± 209	-	41.4 ± 1.0	3.1 ± 0.3	0.780 ± 0.132	6.5
GNP-12	4450 ± 224	-	42.4 ± 1.7	2.5 ± 0.3	0.645 ± 0.115	7.7
GNP-16	5072 ± 270	-	41.6 ± 1.1	2.0 ± 0.1	0.491 ± 0.032	7.4
GNP-20	5725 ± 308	-	42.9 ± 1.6	1.9 ± 0.1	0.468 ± 0.041	7.4
GNP-30	7362 ± 569	-	44.3 ± 1.9	1.3 ± 0.1	0.340 ± 0.034	7.3
CNT-2	2513 ± 101	43.3 ± 0.4	34.1 ± 1.5	7.5 ± 2.4	2.313 ± 0.832	4.3
CNT-4	2622 ± 29	43.5 ± 1.0	40.3 ± 1.8	4.5 ± 0.6	1.253 ± 0.263	3.3
CNT-6	2849 ± 70	-	46.6 ± 0.5	3.9 ± 0.2	1.112 ± 0.110	3.8
CNT-8	3068 ±156	-	45.1 ± 2.3	3.2 ± 0.3	0.805 ± 0.117	4.1

^1^ Normalized value of the improvement of the modulus following Equation (3); ^2^ not defined.

**Table 3:** Creep compliance data of ABS-graphene and ABS-CNT nanocomposites according Equation (17).

Samples	*D_el_* (GPa^−1^)	*D_ve_*_,3600s_ (GPa^−1^)	*D_tot_*_,3600s_ (GPa^−1^)	*D_e_* (GPa^−1^)	*k* (GPa^−1^ s^−n^)	*n*	R^2^
ABS	0.572	0.205	0.777	0.553	0.0164	0.324	0.998
GNP-2	0.451	0.122	0.573	0.448	0.0030	0.464	0.987
GNP-4	0.415	0.084	0.499	0.414	0.0018	0.487	0.982
GNP-6	0.382	0.065	0.447	0.377	0.0077	0.277	0.991
GNP-8	0.328	0.088	0.416	0.329	0.0011	0.536	0.997
GNP-12	0.214	0.077	0.290	0.230	0.0009	0.517	0.991
GNP-30	0.138	0.039	0.177	0.139	0.0002	0.656	0.983
CNT-2	0.515	0.205	0.777	0.502	0.0084	0.373	0.992
CNT-4	0.478	0.152	0.667	0.466	0.0101	0.346	0.994
CNT-6	0.436	0.149	0.627	0.435	0.0033	0.462	0.995
CNT-8	0.413	0.135	0.572	0.408	0.0038	0.455	0.991

**Table 4:** Creep compliance data of ABS-graphene and ABS-CNT nanocomposites at different temperatures according Equation (17).

Samples	*D_el_* (GPa^−1^)	*D_ve_*_,3600s_ (GPa^−1^)	*D_tot_*_,3600s_ (GPa^−1^)	*D_e_* (GPa^−1^)	*k* (GPa^−1^ s^−n^)	*n*	R^2^
T= 50 °C							
ABS	0.604	0.380	0.984	0.5545	0.0164	0.3538	0.982
GNP-6	0.494	0.349	0.843	0.4519	0.0232	0.3553	0.984
CNT-6	0.521	0.334	0.855	0.4711	0.0268	0.3367	0.979
T= 70 °C							
ABS	0.617	0.957	1.574	0.5416	0.0342	0.4243	0.990
GNP-6	0.442	0.836	1.278	0.3436	0.0522	0.3620	0.986
CNT-6	0.534	0.997	1.532	0.4298	0.0531	0.3788	0.989
T= 90 °C							
ABS	0.687	5.747	6.435	0.4931	0.0569	0.5744	0.994
GNP-6	0.516	3.726	4.242	0.3745	0.0507	0.5363	0.994
CNT-6	0.561	3.920	4.482	0.3149	0.0894	0.4774	0.990

**Table 5:** The activation energy for the creep process.

Samples	*E_act_* (kJ/mol)
ABS	15.1 ± 3.4
GNP-6	22.8 ± 1.9
CNT-6	21.2 ± 3.0

## Supplementary Materials

The following are available online at http://www.mdpi.com/2079-4991/8/9/674/s1, Figure S1: Representative stress-strain curves of ABS and ABS nanocomposites with (a) graphene and (b) carbon nanotubes, Figure S2: Creep compliance curves of (a) ABS matrix and nanocomposites with (b) GNP-6 or (c) CNT-6 under applied load of 3.9 MPa at different temperatures in the range 30–90 °C, Figure S3: Comparison of the parameters *P_E_*_,_*_ρ_* encompassing the effects of elastic modulus and resistivity of nanocomposites, as function of the nanofiller fraction up to 8 wt % (see Equation (21)), Figure S4: Comparison of parameters P_E,M,_***_ρ_*** encompassing the effects of elastic modulus, melt flow index (at 220 °C and 10 kg) and resistivity, as function of nanofiller fraction up to 8 wt % of CNT or 30 wt % of GNP (See Equation (22)), Table S1: Summary of representative properties of ABS matrix and its composites with GNP-M5 or CNT nanofillers. The values for filler fractions 2 wt % and 8 wt % are calculated by using Equations (S1) and (S2).
