# Supplementary material for: Effects of the Nanofillers on Physical Properties of Acrylonitrile-Butadiene-Styrene Nanocomposites: Comparison of Graphene Nanoplatelets and Multiwall Carbon Nanotubes
Source: Nanomaterials (Basel). 2018 Aug 29;8(9):674. doi: 10.3390/nano8090674 (PMC6164247; doi:10.3390/nano8090674)
Supplement: Supplementary file 1 [file nanomaterials-08-00674-s001.pdf]

Supplementary Materials: Effects of the nanofillers on physical properties of ABS nanocomposites - Comparison of graphene nanoplatelets and multiwall carbon nanotubes

Sithiprumnea Dul, Alessandro Pegoretti and Luca Fambri*

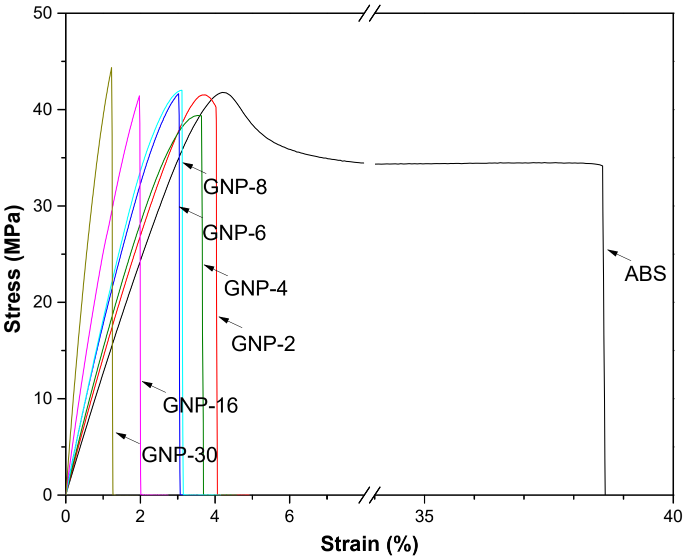

(**a**)

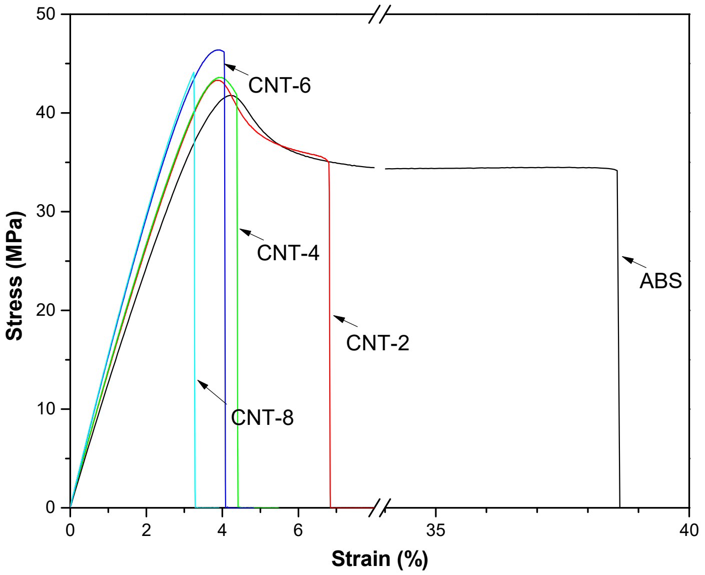

(**b**)

Figure S1. Representative stress-strain curves of ABS and ABS nanocomposites with (a) graphene and (b) carbon nanotubes.

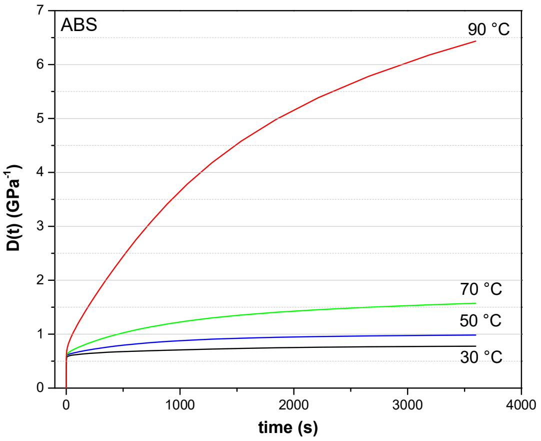

(a)

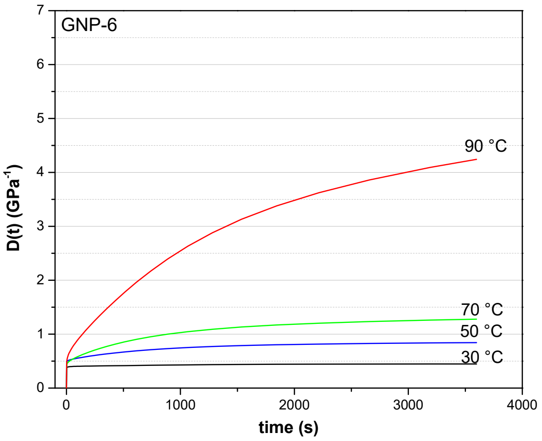

(b)

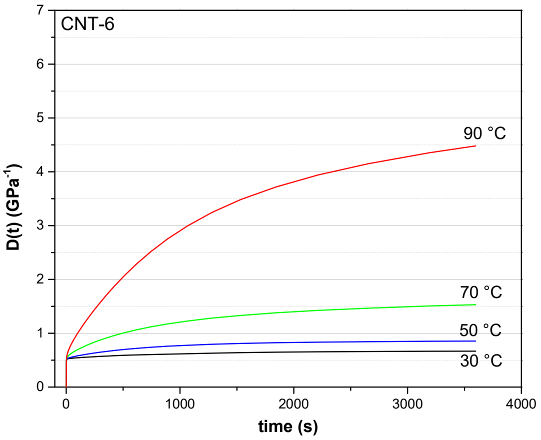

(c)

Figure S2. Creep compliance curves of (a) ABS matrix and nanocomposites with (b) GNP-6 or (c) CNT-6 under applied load of 3.9 MPa at different temperatures in the range 30-90 °C.

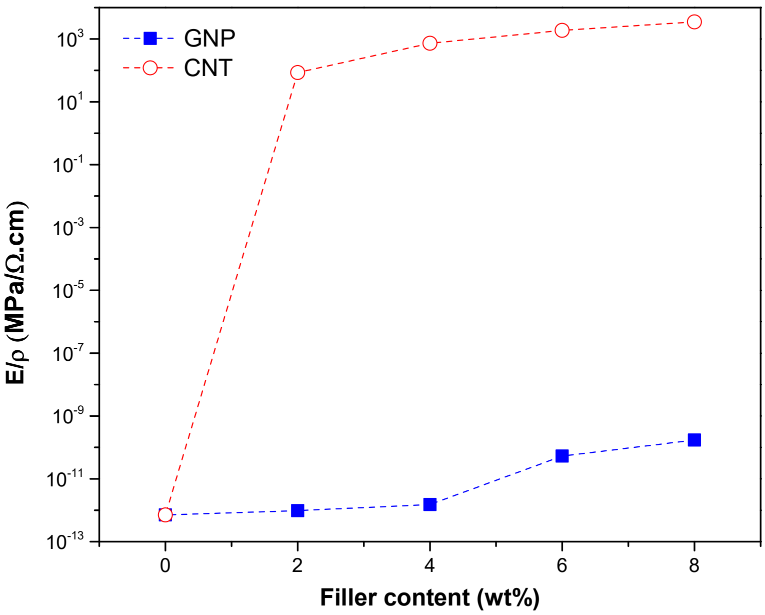

Figure S3. Comparison of the parameters $P_{E,\rho}$ encompassing the effects of elastic modulus and resistivity of nanocomposites, as function of the nanofiller fraction up to 8 wt% (See Eq. 21).

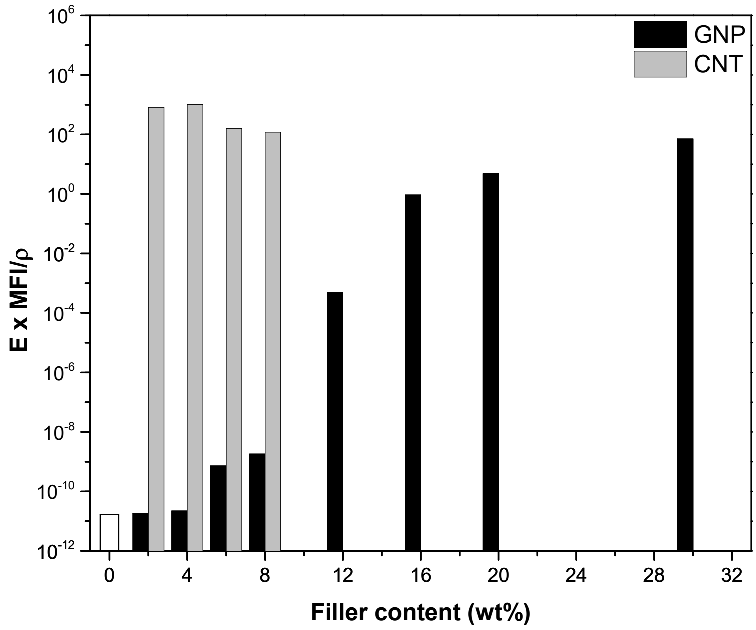

Figure S4. Comparison of parameters $P_{E,M,\rho}$ encompassing the effects of elastic modulus, melt flow index (at 220°C and 10kg) and resistivity, as function of nanofiller fraction up to 8 wt% of CNT or 30 wt% of GNP (See Eq. 22).

Table S1. Summary of representative properties of ABS matrix and its composites with GNP-M5 or CNT nanofillers. The Relative Factors for filler fractions 2 wt% and 8 wt% are calculated by using Eq. (S1) and Eq (S2).

Properties	ABS	GNP-2 ➔ GNP-8	CNT-2 ➔ CNT-8	GNP Relative Factor	CNT Relative Factor	Effective filler
MFI (g/10min) at 250°C	89.7	72.5 ➔ 47.1	39.3 ➔ 0.1	0.81 ➔ 0.53	0.44 ➔ 1.1E-03	GNP
E (MPa)	2315	2631 ➔ 3523	2513 ➔ 3068	1.14 ➔ 1.52	1.09 ➔ 1.33	GNP
Strength (MPa)	41.7	41.5 ➔ 41.4	43.3 ➔ 45.1	1.00 ➔ 0.99	1.04 ➔ 1.08	GNP/CNT
TEB ($MJ.mm^{-3}$)	11.8	1.057 ➔ 0.780	2.313 ➔ 0.805	0.090 ➔ 0.066	0.196 ➔ 0.068	GNP/CNT
Strain at break (%)	35.9	4.1 ➔ 3.2	7.5 ➔ 3.1	0.11➔ 0.09	0.21 ➔ 0.09	GNP/CNT
Creep compliance (GPa^{-1})	0.777	0.573 ➔ 0.416	0.667 ➔ 0.553	0.74 ➔ 0.54	0.86 ➔ 0.71	GNP
Resistivity (Ω.cm)	3.27E+15	2.72E+15 ➔ 2.07E+13	2.94E+01➔ 8.73E-01	1.2E+00 ➔ 1.6E+02	1.1E+14 ➔ 3.7E+15	CNT

The relative factors, GNP Factor and CNT Factor, were calculated as the ratio of a given property of composite (P_C) and of ABS matrix (P_{ABS}) by means of Eq. (S1):

$$\text{Relative Factor} = P_C / P_{ABS} \quad \text{(S1)},$$

where C is GNP or CNT.

In the case of resistivity, the Relative Factor (ρ) was calculated according to the inverse of properties, according to Eq. (S2)

$$\text{Relative Factor } (\rho) = P_{ABS} / P_C \quad \text{(S2)}$$

Data in Table S1 represent the experimental results of the matrix and of the nanocomposites, and the corresponding calculated Relative Factors from the maximum to minimum (or vice versa in the case of modulus and resistivity). It is evident that volume fraction GNP and CNT have similar effects on strength, strain at break and tensile energy to break. On the other hand, GNP is preferred for the improvement effects in stiffening (modulus, creep compliance) and the satisfactory behaviour in processing, whereas CNT could be positively considered for the beneficial effect on resistivity, only.
